# Trial of labor following cesarean among patients with oligohydramnios at term: A multicenter retrospective study

**DOI:** 10.1002/ijgo.70202

**Published:** 2025-05-16

**Authors:** Ari Weiss, Tzuria Peled, Reut Rotem, Hen Y. Sela, Sorina Grisaru‐Granovsky, Misgav Rottenstreich

**Affiliations:** ^1^ Department of Obstetrics and Gynecology, Shaare Zedek Medical Center Affiliated with the Hebrew University School of Medicine Jerusalem Israel; ^2^ Department of Nursing Jerusalem College of Technology Jerusalem Israel

**Keywords:** amniotic fluid, maternal outcomes, neonatal outcomes, oligohydramnios, planned repeat cesarean delivery, trial of labor after cesarean, uterine rupture

## Abstract

**Objective:**

The aim of this study was to evaluate the maternal and neonatal outcomes of patients with oligohydramnios attempting a trial of labor after cesarean (TOLAC) versus those who underwent planned repeat cesarean delivery (PRCD).

**Methods:**

We conducted a multicenter retrospective cohort study of patients with a term singleton pregnancy following a single low‐segment transverse cesarean delivery (CD) and a recent diagnosis of oligohydramnios (maximal vertical pocket <2 cm) between 2017 and 2021. Maternal and neonatal outcomes were compared between patients attempting TOLAC and those opting for PRCD. Univariate analysis was conducted, followed by a multivariate analysis.

**Results:**

A total of 352 deliveries were included, of which 278 (79%) attempted TOLAC and 74 (21%) attempted PRCD. The successful vaginal delivery rate for patients who attempted TOLAC was 84.5%. The uterine rupture rate was not significantly different between those attempted TOLAC versus PRCD (1.1% vs. 0%, *P* = 0.371). However, the rate of hypoglycemia (2.2% vs. 8.1%, *P* = 0.012) and the composite adverse neonatal outcome was higher among patients with PRCD (11.5% vs. 24.3%, *P* = 0.005). After controlling for potential confounders, we still demonstrated an independent association between reduced rates of composite adverse neonatal outcome and TOLAC (adjusted odds ratio 0.46, 95% CI: 0.23–0.92, *P* = 0.028).

**Conclusion:**

TOLAC for patients with oligohydramnios appears to be a reasonable alternative and is associated with favorable outcomes. Further large and prospective research on this subject may lead to improved management strategies and better maternal and neonatal outcomes.

## 1 INTRODUCTION

Oligohydramnios, defined as a deficiency in amniotic fluid, is considered a significant factor contributing to adverse perinatal outcomes, encompassing low Apgar scores, birth asphyxia, cesarean deliveries (CDs), neonatal intensive care unit (NICU) admission, meconium aspiration syndrome, and respiratory distress syndrome.^1^, ^2^, ^3^, ^4^, ^5^, ^6^, ^7^, ^8^

In the context of patients with a history of previous CD, there is a global initiative to minimize repeated CDs due to associated morbidity. Trial of labor after cesarean (TOLAC) has emerged as a practical and widely endorsed option by various formal organizations and guidelines, aiming to reduce the overall incidence of CDs and their repeats.^9^, ^10^, ^11^ However, the efficacy in decreasing complications lies not only in promoting TOLAC but also in judiciously selecting appropriate candidates, as a failed TOLAC might lead to urgent CD, and carries an elevated risk of adverse maternal and neonatal outcomes.^12^, ^13^, ^14^

Despite previous studies that have reported an increased risk of CD in cases of isolated or uncomplicated oligohydramnios,^15^, ^16^ data regarding TOLAC outcomes in this subgroup of patients with oligohydramnios diagnosed at term is limited.

The present study aimed to assess the maternal and neonatal outcomes among patients diagnosed with oligohydramnios at term who underwent TOLAC compared to those who underwent planned repeat cesarean delivery (PRCD), thus providing comprehensive insights that might help clinical decision making of optimal delivery strategies in this specific subgroup of patients.

## 2 MATERIALS AND METHODS

A multicenter retrospective cohort study was conducted using electronic medical records from two university‐affiliated medical centers in Jerusalem, Israel—Shaare Zedek Medical Center (SZMC) and Bikur Holim Medical Center (BHMC), spanning the years 2017 to 2021. These centers collectively account for approximately 16% of all deliveries in Israel, averaging 22 000 births annually during the study period.

Although oligohydramnios is usually defined as an amniotic fluid index (AFI) of ≤5 cm or a maximum vertical pocket (MVP) of <2 cm measured by ultrasound,^17^ as AFI overcalls oligohydramnios leading to more interventions without any improvement in outcomes,^18^, ^19^ for the purpose of the present study our criteria was restricted to cases in which oligohydramnios diagnosed by MVP was<2 cm.^20^

The study included term (37–42 weeks) singleton viable pregnancies with a live fetus attempting the first trial of labor following a single low‐segment transverse CD, with a diagnosis of oligohydramnios. In all cases the diagnosis was given or confirmed by an ultrasound closes to delivery upon admission to Labor and Delivery. We excluded patients in which the oligohydramnios was resolved, patients with AFI <5 cm but MVP >2 cm, patients with two or more previous CDs, multifetal gestation, placenta‐accreta spectrum, out‐of‐hospital deliveries, malformed uterus fetal major malformations, and non‐vertex presentations.

Both SZMC and BHMC followed shared departmental protocols adhering to the TOLAC guidelines established by the Israeli Committee of Obstetrics and Gynecology which are aligned with the American College of Obstetricians and Gynecologists (ACOG) guidelines.^21^ TOLAC was offered to those with previous single low‐segment transverse CD after a detailed consultation on the risks and benefits of TOLAC versus PRCD, and patients who attempted TOLAC signed an informed consent. Resident physicians managed TOLAC deliveries under the supervision of a board‐certified obstetrician, ensuring real‐time decisions on induction, labor augmentation, operative deliveries, and emergency CDs. Continuous electronic fetal monitoring was mandatory. Regarding the management of oligohydramnios, according to the Israeli guidelines, isolated oligohydramnios at term without additional signs of placental insufficiency, is not an indication for induction of labor. The decision between delivery and conservative monitoring is based on gestational age and clinical judgment. Delivery is considered in appearance of additional signs of placental insufficiency (IUGR, Doppler abnormalities), maximum amniotic fluid pocket less than 2 cm, any suspicion of fetal compromise, and 41 weeks' gestation or more. The mode of delivery is determined based on cervical conditions and obstetric history. Prostaglandins, balloon catheter, and oxytocin may be used as in other standard indications for labor induction.^22^

The study received approval from SZMC's institutional ethics committee (IRB: 0346‐22‐SZMC) overseeing both medical centers' research activities, adhering to the Declaration of Helsinki. Given its retrospective and de‐identified nature, informed consent was waived. Demographic and obstetric data, along with delivery complications, were extracted from the electronic database to minimize retrospective study biases.

### 2.1 Study outcomes

Maternal and neonatal outcomes were compared between TOLAC and PRCD groups. The primary outcome was maternal composite adverse outcome, comprising one or more of the following: uterine rupture, postpartum hemorrhage, blood product transfusion, hysterectomy, laparotomy, puerperal fever, maternal ICU admissions, or prolonged hospitalization (≥7 days after cesarean, >5 days after vaginal delivery). Secondary outcomes included the rate of successful vaginal birth after cesarean (VBAC) among the TOLAC group and various maternal and neonatal outcomes.

### 2.2 Statistical analysis

Statistical analyses were performed using SPSS software (version 25, IBM, Armonk, NY). Descriptive statistics were used for categorical and continuous variables. Chi‐square or Fisher exact tests were employed for categorical variables, while unpaired student's *t*‐test or Mann–Whitney *U* test was used for continuous variables, depending on their distribution. Univariate analysis tested associations between TOLAC and PRCD and various maternal demographic, obstetric, and delivery characteristics. All analyses were two‐sided, and a *P* value of less than 0.05 was considered statistically significant.

Variables showing significance in univariate analysis for primary and secondary outcomes were included in a multivariable logistic regression model. This model assessed the association between TOLAC versus PRCD and the primary and secondary outcomes. Adjusted odds ratios (aOR) with 95% confidence intervals (CIs) were reported for these analyses.

## 3 RESULTS

**FIGURE 1:**
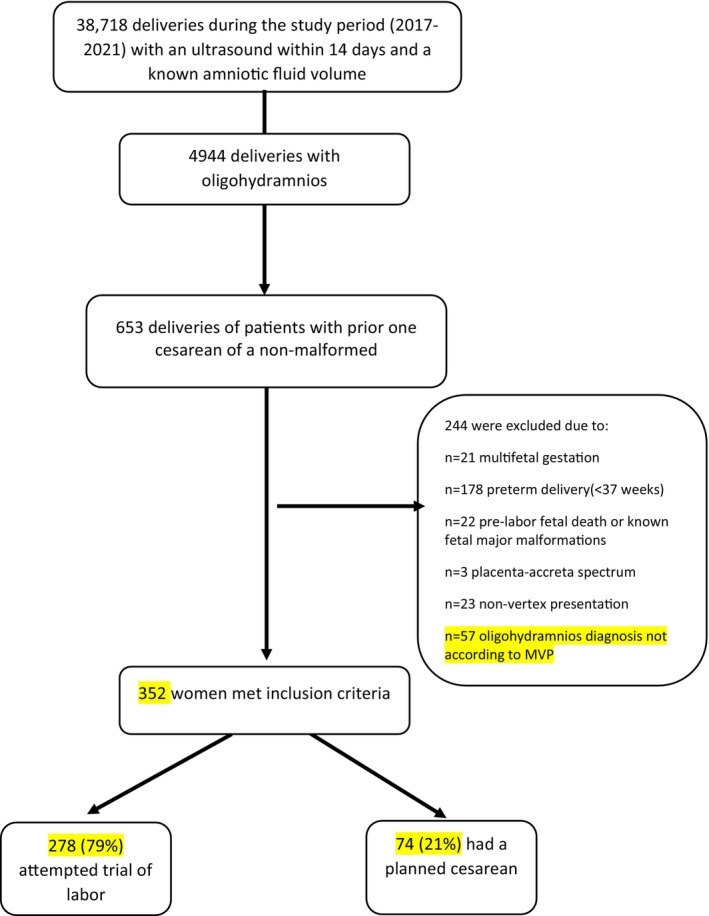
Study population schematic flow chart.

Over the study duration, 352 term deliveries with oligohydramnios in patients with previous CD met the inclusion criteria. Among them, 278 (79%) chose to attempt a TOLAC, while 74 (21%) preferred a PRCD, as illustrated in Figure 1.

Demographic and obstetrical characteristics of the study groups are delineated in Table 1. Notably, those attempting TOLAC were younger (mean age 32.2 ± 5.6 vs. 33.7 ± 5, *P* = 0.038), at advanced gestational age (39.7 ± 1.2 vs. 39 ± 1.6, *P* < 0.001), and delivered larger birth weight neonates (3206.5 ± 475.9 vs. 3063.4 ± 538, *P* = 0.026). However, the incidence of small for gestational age neonates was comparable between the TOLAC and PRCD groups. Table 2 presents the maternal outcomes of both groups.

**TABLE 1:** Baseline and labor characteristics of the study population.

	Planned repeat cesarean *n* = 74	Trial of labor *n* = 278	*P* value
Maternal age, years	33.7 ± 5	32.2 ± 5.6	0.038
Previous miscarriages, any	32 (43.2%)	100 (36%)	0.252
Gravidity	5.3 ± 3.6	5.2 ± 3	0.829
Parity	4.4 ± 3.1	4.5 ± 2.5	0.869
Fertility treatments	6 (8.1%)	20 (7.2%)	0.790
Hypertensive disorders of pregnancy	4 (5.4%)	7 (2.5%)	0.206
Smoking	1 (1.5%)	5 (1.9%)	0.816
Diabetes (pregestational and gestational)	8 (10.8%)	10 (3.6%)	0.012
Obesity (BMI ≥30)	18 (29.5%)	58 (25.3%)	0.511
Gestational age at delivery	39 ± 1.6	39.7 ± 1.2	<0.001
Birth weight, grams	3063.4 ± 538	3206.5 ± 475.9	0.026
Small for gestational age	10 (32.3%)	13 (16.7%)	0.073

*Note*: BMI, calculated as weight in kilograms divided by the square of height in meters. Data are mean ± standard deviation; number (%).

Abbreviation: BMI, body mass index.

**TABLE 2:** Obstetric maternal outcomes among the study groups.

	Planned repeat cesarean *n* = 74	Trial of labor *n* = 278	*P* value
Primary outcomes			
Composite adverse maternal outcome^a^	8 (10.8%)	40 (14.4%)	0.427
Secondary outcomes			
Unplanned cesarean	7 (9.5%)	43 (15.5%)	0.189
Uterine rupture	0 (0%)	3 (1.1%)	0.371
Hysterectomy	0 (0%)	0 (0%)	N/A
Postpartum hemorrhage	7 (9.5%)	36 (12.9%)	0.417
Blood products transfusion	0 (0%)	0 (0%)	N/A
Puerperal fever	1 (3.2%)	2 (2.6%)	0.851
Laparotomy	0 (0%)	0 (0%)	N/A
Maternal ICU admissions	0 (0%)	0 (0%)	N/A
Prolonged hospital stays^b^	0 (0%)	1 (1.3%)	0.531

*Note*: Data are mean ± standard deviation; number (%).

Abbreviation: ICU, intensive care unit.

^a^ A composite adverse maternal outcome including at least one of the following: postpartum hemorrhage, blood products transfusion, maternal ICU admissions, prolonged hospitalization, puerperal fever, uterine rupture, laparotomy, and hysterectomy.

^b^ Hospitalization >7 days after cesarean >5 days after vaginal delivery.

### 3.1 Primary outcomes

The rates of composite adverse maternal outcomes were similar between individuals who attempted TOLAC and those who underwent PRCD (40 [14.4%] vs. 8 [10.8%], *P* = 0.427).

### 3.2 Secondary outcomes

Among patients with oligohydramnios attempting TOLAC, the rate of successful vaginal delivery was 84.5% (235 women) and 43 underwent unplanned CD (15.5%). Of those 25 underwent CD due to suspected fetal distress. Among those with PRCD, seven patients (9.5%) arrived earlier with delivery symptoms and underwent an unplanned CD. Uterine rupture occurred among those who attempted TOLAC in three cases (1.1%), with no cases observed in the PRCD group, but this was not statistically significant (*P* = 0.371). Importantly, there were no occurrences of hysterectomy, laparotomy, or maternal ICU admissions in the study cohort. Rates of postpartum hemorrhage, puerperal fever, blood product transfusion, and prolonged hospital stays were comparable between the two groups.

Table 3 presents the neonatal outcomes of both groups. Even though most neonatal morbidities were not statistically significantly different between the groups, the rate of composite adverse neonatal outcome was higher among patients who underwent PRCD (18 [24.3%] vs. 32 [11.5%], *P* = 0.005).

**TABLE 3:** Neonatal outcomes among the study groups.

	Planned repeat cesarean *n* = 74	Trial of labor *n* = 278	*P* value
5‐min Apgar score <7	2 (2.7%)	1 (0.4%)	0.052
NICU admission	4 (5.5%)	13 (4.7%)	0.777
Prolonged neonatal admission^a^	3 (4.1%)	12 (4.3%)	0.921
Meconium aspiration syndrome	0 (0%)	0 (0%)	N/A
Jaundice	5 (6.8%)	9 (3.2%)	0.170
TTN	0 (0%)	2 (0.7%)	0.466
Mechanical ventilation	0 (0%)	3 (1.1%)	0.371
Seizures	0 (0%)	2 (0.7%)	0.466
Hypoglycemia	6 (8.1%)	6 (2.2%)	0.012
Intracranial hemorrhage	0 (0%)	1 (0.4%)	0.607
Birth asphyxia	0 (0%)	3 (1.1%)	0.371
Composite adverse neonatal outcome^b^	18 (24.3%)	32 (11.5%)	0.005

*Note*: Data are mean ± standard deviation; number (%).

Abbreviation: NICU, neonatal intensive‐care unit; TTN, transient tachypnea of the newborn.

^a^ Prolonged neonatal admission >5 days for vaginal delivery or >7 days for cesarean delivery.

^b^ Composite adverse neonatal outcome—at least one of the above neonatal outcomes.

To assess the association between TOLAC and composite adverse maternal and neonatal outcomes while considering significant covariates and confounders, multiple adjusted multivariable logistic regression analyses were performed. This analysis revealed that TOLAC was not independently associated with composite adverse maternal outcomes (adjusted odds ratio [aOR] 1.14, 95% confidence interval [CI]: 0.49–2.63, *P* = 0.759), but it was associated with decreased rates of composite adverse neonatal outcomes (aOR 0.46, 95% CI: 0.23–0.92, *P* = 0.028), Table 4.

**TABLE 4:** Multivariate logistic regression analysis for the association between TOLAC and composite adverse maternal and neonatal outcomes.

	*P* value	aOR	95% CI	
Composite adverse maternal outcome^a^	0.759	1.14	0.49	2.63
Composite adverse neonatal outcome^b^	0.028	0.46	0.23	0.92

Abbreviations: aOR, adjusted odds ratio; CI, confidence interval; TOLAC, trial of labor after cesarean.

^a^ Adjusted for significant covariates in univariate analysis maternal age, hypertensive disorders of pregnancy, gestational age at delivery, and neonatal birth weight.

^b^ Adjusted for significant covariates in univariate analysis: hypertensive disorders of pregnancy, gestational age at delivery, fertility treatments, and neonatal birth weight.

## 4 DISCUSSION

### 4.1 Principal findings

TOLAC following one previous CD is widely accepted and generally preferred over repeated CD to reduce the complications associated with multiple CDs.^21^, ^23^, ^24^ However, the safety and success rates of TOLAC in pregnancies diagnosed with oligohydramnios are not well‐established. In this multicenter retrospective study of patients with a recent diagnosis of oligohydramnios, we observed a relatively high TOLAC success rate of 84.5%. Univariate analysis indicated that the rate of composite adverse maternal outcomes was similar between patients who attempted TOLAC and those who underwent PRCD. However, the rate of composite adverse neonatal outcomes was higher among patients who underwent PRCD. After adjusting for potential confounders in a multivariable logistic regression, TOLAC was found to be independently associated with decreased rates of composite adverse neonatal outcomes but with no independent association to composite adverse maternal outcomes.

### 4.2 Results in the context of prior studies

In the general population of patients with a history of CD, the TOLAC success rate is estimated to be between 50% and 90%, depending on various demographic and obstetrical factors.^21^, ^25^, ^26^, ^27^, ^28^ The success rate and adverse outcomes of TOLAC in pregnancies complicated by oligohydramnios have not been extensively studied. Our literature review revealed no studies directly addressing this issue. However, some studies have assessed TOLAC in subgroups, including pregnancies complicated by oligohydramnios.

In a study by Ouzounian et al.,^29^ the authors examined the outcome of intrapartum amnioinfusion. Among 936 patients, 76.6% underwent amnioinfusion due to oligohydramnios, with the remainder undergoing amnioinfusion due to meconium‐stained amniotic fluid or variable decelerations. Of the patients who underwent amnioinfusion, 122 had a previous CD and attempted TOLAC. The TOLAC success rate in this group was 58.2%, compared to 76.5% for those with no history of CD. Uterine rupture rates were not significantly different between patients who underwent amnioinfusion and those who did not (0.8% vs. 1.1%, *P* = 0.6). However, specific data regarding the oligohydramnios subgroup in the TOLAC cohort was missing.

Another relevant study by Gregory et al.,^30^ a population‐based cohort study of 41 450 patients with a history of CD who attempted TOLAC, demonstrated a 50% success rate for TOLAC in pregnancies with oligohydramnios. The study noted that the nationally reported VBAC rate for patients with oligohydramnios was 19%. This study compared the oligohydramnios subgroup to patients with different conditions who attempted TOLAC but did not compare them to women with oligohydramnios who underwent PRCD. It was found that, compared to all patients without oligohydramnios, oligohydramnios in patients attempting TOLAC was associated with significantly higher rates of postpartum hemorrhage (PPH), longer maternal and neonatal hospitalization, and higher rates of neonatal adverse outcomes, including hypoxia, infection, and seizures. However, they did not demonstrate a higher rate of uterine rupture.

In our study, we had a relatively high TOLAC success rate of 84.5%. This high success rate and favorable safety profile could be attributed to the extensive experience and specialized care provided in our centers, which manage TOLAC cases regularly and adhere meticulously to departmental protocols allowing TOLAC.

### 4.3 Clinical implications

Our study demonstrates a relatively high success rate of TOLAC in patients with a diagnosis of oligohydramnios, without an elevated risk of maternal or neonatal outcomes compared to PRCD. Healthcare providers can use this information to counsel patients, providing reassurance about the likelihood and safety of a successful TOLAC. While this study assessed the short‐term outcomes of patients with oligohydramnios who underwent TOLAC versus PRCD, repeated CDs are associated with long‐term complications in subsequent pregnancies, such as placenta previa, placenta accreta, placental abruption, adhesions, bladder and bowel injury, excessive bleeding, blood transfusions, and hysterectomy.^21^, ^31^, ^32^ These complications should be considered when consulting patients with oligohydramnios and a previous CD about the mode of delivery and may tip the scales toward TOLAC when eligible.

### 4.4 Research implications

This study contributes to the existing literature on TOLAC by establishing high success rates and satisfactory maternal and neonatal outcomes in patients with a recent diagnosis of oligohydramnios. Although we demonstrated lower rates of adverse neonatal outcomes in patients who underwent TOLAC compared to PRCD, this was not statistically significant in multivariate analysis. This study serves as a steppingstone for future investigations, encouraging the development of targeted strategies to improve maternal and neonatal outcomes in patients with oligohydramnios undergoing TOLAC. Further larger studies are needed to confirm our findings. Additionally, future studies should focus on TOLAC in other specific subgroups of patients with oligohydramnios, such as those with preterm premature rupture of membranes (PPROM), pre‐eclampsia, post‐term pregnancies, and fetuses with fetal growth restriction (FGR), as the sample size of our study was too small to assess these groups. This will help establish safety and explore predictive factors and potential interventions that can optimize TOLAC success rates in these specific subgroups.

In the present study, we chose to evaluate the outcomes of patients with oligohydramnios and one previous CD by comparing TOLAC to PRCD, rather than comparing patients with oligohydramnios who underwent TOLAC to those with normal amniotic fluid levels. This approach was intended to better inform our consultations with patients facing this concern. Although choosing a control group of patients with normal fluid levels would be academically interesting and add information about the additive risk of oligohydramnios itself, it does not address the clinical dilemma.

### 4.5 Strengths and limitations

The main strength of our study was the large cohort from two obstetrical centers with high annual delivery volumes, in a developed country with advanced medical services and relatively high rates of TOLAC attempts. This enabled us to evaluate this specific subgroup of patients with oligohydramnios. The uniformity in inclusion criteria, reflected by the strict definition of oligohydramnios only according to one method (MVP) and the timing of diagnosis by qualified ultrasound units within the hospital, along with consistent TOLAC management protocols in our centers, reduces the likelihood of confounders and increases the reliability and generalizability of our findings. The use of computerized medical records updated in real‐time provided comprehensive data and minimized bias.

Our study specifically assessed success rates and complications of TOLAC in the presence of oligohydramnios. Additionally, the diagnosis of oligohydramnios in our study was close to delivery, whereas, in the previous studies discussed above, the timing of diagnosis was not specified and relied on medical record ICD codes.

The main limitation of our study was its retrospective design, which carries inherent limitations. For instance, we did not have sufficient data to stratify according to the causes of oligohydramnios or the indications for the previous CD, which could be important factors in successful TOLAC.^33^ Additionally, the unique population in our centers, characterized by patients who tend toward large families and prefer vaginal delivery over CD, may influence both the physicians' and patients' preferences regarding the mode of delivery. Despite these limitations, we addressed this through the inclusion of a large sample size and comparison of our results with other studies conducted in different populations that showed similar success rates. The rates of maternal and neonatal complications were similar to those reported in previous worldwide publications, including uterine rupture, PPH, puerperal fever, maternal ICU admissions, and maternal respiratory complications.^21^, ^34^ This similarity suggests that our study's conclusions may be generalizable to other populations.

## 5 CONCLUSIONS

In this multicenter study of patients with oligohydramnios, we found that the successful vaginal delivery rate for those who attempted TOLAC was 84.5%. We also compared the maternal and neonatal outcomes of patients with oligohydramnios between those who underwent TOLAC versus those who underwent PRCD, demonstrating similar maternal outcomes between the groups, with no increased risk of uterine rupture. The rate of hypoglycemia and composite adverse neonatal outcomes was higher among patients who underwent PRCD, and this association remained significant after even controlling for potential confounders. This relatively high success rate demonstrates that under the right conditions, TOLAC in pregnancies complicated by oligohydramnios is an achievable and safe option, with potentially favorable outcomes.

These new findings enhance our understanding of this obstetric condition and may increase the rates of TOLAC in this subpopulation, thereby reducing the rates of PRCD and its associated early and late complications. Further studies from other medical centers are needed to strengthen these findings.

## AUTHOR CONTRIBUTIONS

AW designed, planned, and conducted the study, and wrote the manuscript, TP designed, planned, and conducted the study and wrote the manuscript, RR designed, planned, and conducted the study and wrote the manuscript, HYS conducted the study and edited the manuscript, SGG analyzed the data and edited the manuscript, and MR designed, planned and conducted the study.

## CONFLICT OF INTEREST STATEMENT

None of the authors declare any conflict of interest.

## Data Availability

Research data are not shared.
